# Identification of Candidate Small-Molecule Therapeutics to Cancer by Gene-Signature Perturbation in Connectivity Mapping

**DOI:** 10.1371/journal.pone.0016382

**Published:** 2011-01-31

**Authors:** Darragh G. McArt, Shu-Dong Zhang

**Affiliations:** Centre for Cancer Research and Cell Biology (CCRCB), Queen's University Belfast, Belfast, United Kingdom; Fondazione Telethon, Italy

## Abstract

Connectivity mapping is a recently developed technique for discovering the underlying connections between different biological states based on gene-expression similarities. The sscMap method has been shown to provide enhanced sensitivity in mapping meaningful connections leading to testable biological hypotheses and in identifying drug candidates with particular pharmacological and/or toxicological properties. Challenges remain, however, as to how to prioritise the large number of discovered connections in an unbiased manner such that the success rate of any following-up investigation can be maximised. We introduce a new concept, gene-signature perturbation, which aims to test whether an identified connection is stable enough against systematic minor changes (perturbation) to the gene-signature. We applied the perturbation method to three independent datasets obtained from the GEO database: acute myeloid leukemia (AML), cervical cancer, and breast cancer treated with letrozole. We demonstrate that the perturbation approach helps to identify meaningful biological connections which suggest the most relevant candidate drugs. In the case of AML, we found that the prevalent compounds were retinoic acids and PPAR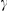 activators. For cervical cancer, our results suggested that potential drugs are likely to involve the EGFR pathway; and with the breast cancer dataset, we identified candidates that are involved in prostaglandin inhibition. Thus the gene-signature perturbation approach added real values to the whole connectivity mapping process, allowing for increased specificity in the identification of possible therapeutic candidates.

## Introduction

Different biological states have their own characteristic gene-expression profiles, and these profiles reflect the state of the cell and offer an insight into possible effectors to influence the phenotype. A feature of microarray gene expression profiles is the ability to distinguish between disease states and that of normal states [1]–[3]. The difference in gene expression can be used to formulate a query gene signature based on pertinent genes retrieved by statistical differentiation. This can form the basis of a disease-gene-drug connection, with the discovery of potential candidate therapeutics. Such a connection between divergent fields has the ability to propose alternative treatments for diseases that are often painstakingly acquired over extended periods of time on a particular known effector/inhibitor [4].

The Connectivity Map concept was first introduced by Lamb *et al.* (2006) as an endeavor to tackle one of the fundamental challenges in biomedicine, to establish the connections between genes, drugs, and diseases [3]. The underlying principle is that gene expression differentiation can adequately characterise a biological state, with which the formulation of a connection between two states can be made by comparing their genomic signatures. The key components of formulating such a connection are 1) a core database of gene expression profiles, 2) a query gene signature, and 3) an algorithm that utilises pattern matching to establish connections [1]. As a further development upon the initial Connectivity Map concept, Zhang and Gant (2008) developed a simpler and unified framework for carrying out the connectivity mapping exercise [5], which was implemented in sscMap (statistically significant connections' map) that offers researchers the ability to make statistically significant connections between query gene signatures (the gene lists to be analysed) and reference profiles (the gene lists used as the reference in the connectivity mapping process) [6].

Connectivity mapping methods, sscMap in particular, allow the possibility of the use of alternative therapeutics and novel applications of existing drugs [3], [5], [7]. The connectivity mapping approach has been applied to studies to discover candidate therapeutics to neuroblastoma and hepatocellular carcinoma [8], [9]. It was a useful resource in these instances as prognosis for these types of cancers are particularly poor. This is an important factor that enhances the potential opportunity offered by these techniques. Other interesting techniques that have developed from the original Connectivity Map concept have looked at addressing the mode of action and molecular effects by way of a “drug network (DN)” [10]. Iorio *et al.* developed a tool to explore the DN and query it with undescribed compounds for classification. Another area looked at by Iskar *et al.* was that of utilising filtering and normalisation steps to improve the signal to noise ratio and elucidate drug-induced feedback mechanisms using the Connectivity Map [11]. sscMap differs from these approaches by exploring the avenue of selecting robust candidates for the potential to alter the phenotype of disease using a modified framework of the original Connectivity Map. Through introducing a new ranking and scoring scheme it provides safeguards and protects against false positives. [5].

The application of a methodology that would allow enhanced sensitivity in therapeutic candidate selection would offer an attractive synergy with the sscMap concept. Through a method of increasing the confidence in therapeutic potential it would shorten the list of retrieved candidates and heighten the chance for successful application. In order to make the best possible connections between these states it is important that the selection process is capable of predicting candidates accurately. The sensitivity and specificity of sscMap ensure the return of statistically significant connections and the accumulation of therapeutic candidates, but it is how these are ranked to maximise their effectiveness is of importance. To achieve this we introduced a new concept, gene-signature perturbation, which helps to discover meaningful biological connections that are robust and stable against systematic small changes (perturbation). Briefly, this test of robustness with connections between gene signatures and reference profiles is obtained by omitting each gene (probeset) once, with replacement, and witnessing its effect on the concurrent listing of hypothesised therapeutic candidates. A perturbation stability score (see the Methods section for detailed definition and procedure) is calculated for each candidate, with a maximum score of 1 indicating that the candidate therapeutic in question was stable and robust against perturbation in the analysis. A decreasing score would indicate that the candidates were not of sufficient caliber to require immediate attention. The foundation of this approach allows the user to have a list of potential candidate compounds that have performed strongly amongst the built-in core database of reference profiles. Extending this approach to understand the therapeutic ‘strength’ in an unbiased way would enhance the utility of sscMap. To assess this we have implemented a method of this perturbation testing in order to rank candidate compounds by their ability to withstand these slight changes to the signature gene list.

We examined this approach on three publicly available datasets from the GEO database [12]. We attained curated microarray datasets for cervical cancer, acute myeloid leukemia and breast cancer (letrozole treated) [13]–[15]. For each case study the raw data were downloaded in Affymetrix CEL format and the relevant information garnered from packages in the Bioconductor suite [16].

Acute myeloid leukemia (AML) is a disease with a particularly poor prognosis. It is a disease where abnormal myeloid blood cells are produced and accumulate in the peripheral blood and bone marrow [17]. AML has high levels of genetic heterogeneity and this makes targeted therapies challenging [18]. The GEO curated dataset (GSE9476, of type HG-U133A) contained 64 samples, 38 from healthy donors and 26 from AML patients. The challenges of surmounting the heterogeneity have led researchers to use high-throughput technology in an attempt to identify genes with abnormal expression for the development of specific and less toxic therapies [14].

Cervical cancer is the second most common type of cancer in women worldwide and the majority of advanced cases detected die [19], [20]. Cervical cancer cells show karyotype alteration complexity and it is thought that characterisation of this genetic variability will help reveal genes paramount to cervical cancer development [13]. The dataset on cervical cancer (GSE9750, of type HG-U133A) contained a total of 66 samples, 24 of which were normal cervical epithelium, 33 primary tumours and 9 cell lines. In our analysis the 9 cell lines were omitted to reduce variability.

Breast cancer is the most common cancer among women. Estrogen hormones are important regulator of physiological processes, and they play a role in breast cancer cell proliferation by binding to their target receptor [21] and therefore offer a therapeutic avenue to treatment by antagonism of estrogens actions [22]. Letrozole, an aromatase inhibitor, was applied to breast cancer patients in the last case study and genetic profiles were examined. The dataset (GSE5462, of type HG-U133A) contained 116 samples in a paired study taking biopsies before and after (10–14 days) letrozole treatment and it offered a different scenario from the first two datasets for the connectivity mapping exercise.

## Results

### Acute Myeloid Leukemia

#### The AML gene signature

Two-sample t-tests (26 AMLs vs 38 Healthy) on the AML dataset returned 186 significant differentially expressed genes with an expected number of false positives (ENFP)<1. This list was then filtered by removing those genes whose mean log2 expression intensities were below 6 in both the healthy and AML groups. The setting of a minimum log2 intensity at 6, although somewhat arbitrary, was based on our previous experience working with Affymetrix arrays, the rationale being that bright probes with fluorescence intensity above certain threshold give more reliable results. This filtering by probe intensity reduced the gene list to 118. Adding a further stringency by analyzing the fold changes, where accepting a threshold >1 for the absolute log2-ratio reduced the list to 105 significant genes. In order to derive a representative gene signature, different lists were compiled with the first 105, 50, 30, 20 and 10, of the top ranked genes by p-value. sscMap analyzed these gene signatures of different length and the 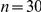 gene signature was determined to be the one that returned a list of candidate compounds of significant length, and was further analyzed by the perturbation method. Creating 30 perturbation signatures based on the original 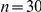 gene signature, these 31 gene signatures together were re-analyzed by sscMap and a perturbation stability of each candidate connection was obtained.

#### The connections to drugs

The AML case study gave five compounds with significant inverse connections to AML (see top of Table 1). Inverse connection here refers to a connection with negative setscore defined by Eq.(6) in [5]. A negative setscore indicates that the biological state collectively represented by the reference set is opposing the biological state represented by the gene signature. In this particular case with AML, it means that these candidate compounds all have potential effects of countering the AML disease state. Each candidate was then ranked by their setsize (SetS) if in the case where the perturbation stabilities (PS) are equal. There was only one candidate with a perturbation stability 1. This suggests that the compound 5186223 was a stable candidate throughout the perturbation experiment even though it had a setszie of 1. The SetSs for the the candidates varied from 4 to 1 which were low but the inverse connections retrieved were relatively strong, represented by negative setscores ranging from −0.3879 to −0.2377.

**Table 1:** Compounds with significant connections to the AML gene signature.

REF	TabNo	SS	PS	SetNo	SetS
5186223	31	31	1	−0.3879	1
Prestwick-691	31	28	0.9032	−0.3437	3
TTNPB	31	17	0.5484	−0.3049	2
co-dergocrine mesilate	31	15	0.4838	−0.2377	4
iloprost	31	12	0.3871	−0.2796	3
neomycin	31	31	1	0.1705	5
dilazep	31	31	1	0.1922	5
tranylcypromine	31	31	1	0.2588	5
solasodine	31	31	1	0.1551	6

Those at the top of the table with negative setscores are candidates that may inhibit the AML phenotype. At the bottom of the table with positive setscores are compounds that may elicit a transcriptional response similar to that of AML. REF is the (drug) name for the reference set; TabNo (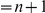) is the total number of gene signatures used in the perturbation analysis; SS is the total number of significant connections to REF from those 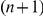 signatures; PS is the perturbation stability 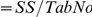, and SetNo is the setscore; SetS is the setsize.

The literature for 5186223 is scarce and Prestwick-691, the next ranked candidate, follows suit with a dearth of information available. The aromatic retinoid TTNPB has been shown to play a key role in decreasing the proliferation of melanoma cells under rexinoid and retinoid treatment [23]. Co-dergocrine mesilate works by stimulating presynaptic dopamine receptors which inhibits norepinephrine secretion, with norepinephrine having been shown to increase the proliferation of some cancers [24]. Iloprost, a synthetic prostacyclin analogue, has been suggested as a candidate therapeutics to cancer. It decreases tumour cell adhesion to endothelium by binding to the prostacyclin receptor and is reported to have antimetastatic effects [25]. All these five compounds have a negative connection score to the AML gene signature, suggesting that they may serve as candidate drugs to treat AML, or more precisely, they all have the potential to push the biological state represented by AML patients back closer to the biological state represented by healthy donors.

Adverse compounds retrieved by the AML case study gave a list of four compounds with a perturbation stability of 1 and a SetS of at least 5, bottom of Table 1 (solasodine, tranylcypromine, dilazep and neomycin). Solasodine is a glycoalkaloid, which may have a role in skin cancer protection [26]; tranylcypromine is a monoamine oxidase inhibitor, with monamine oxidase down-regulation a potential indicator of cancer risk [27]; dilazep is an inhibitor of equilibrative transport of nucleotides [28]; and neomycin is an inhibitor of phospholipase C which has an important role in signal transduction pathways. This list, was again, filtered to include candidates that withstood the perturbation stability test (bottom of Table 1). Candidates were ranked by their PS and then by SetS in decreasing order. SetNos ranged from 0.1551 to 0.2588. The fact that these compounds have positive connections to AML indicate that they can elicit a transcriptional response similar to that observed in AML. Generally, compounds with strong positive connections to a disease signature are not to be used as candidate therapeutics to that disease. However, their known mode of actions could nevertheless provide valuable insights about the mechanisms which are impaired or compromised in the disease state. A comprehensive list of drugs connected to the AML gene signature can be found in the supplementary Table S1.

### Cervical cancer

**Figure 1:**
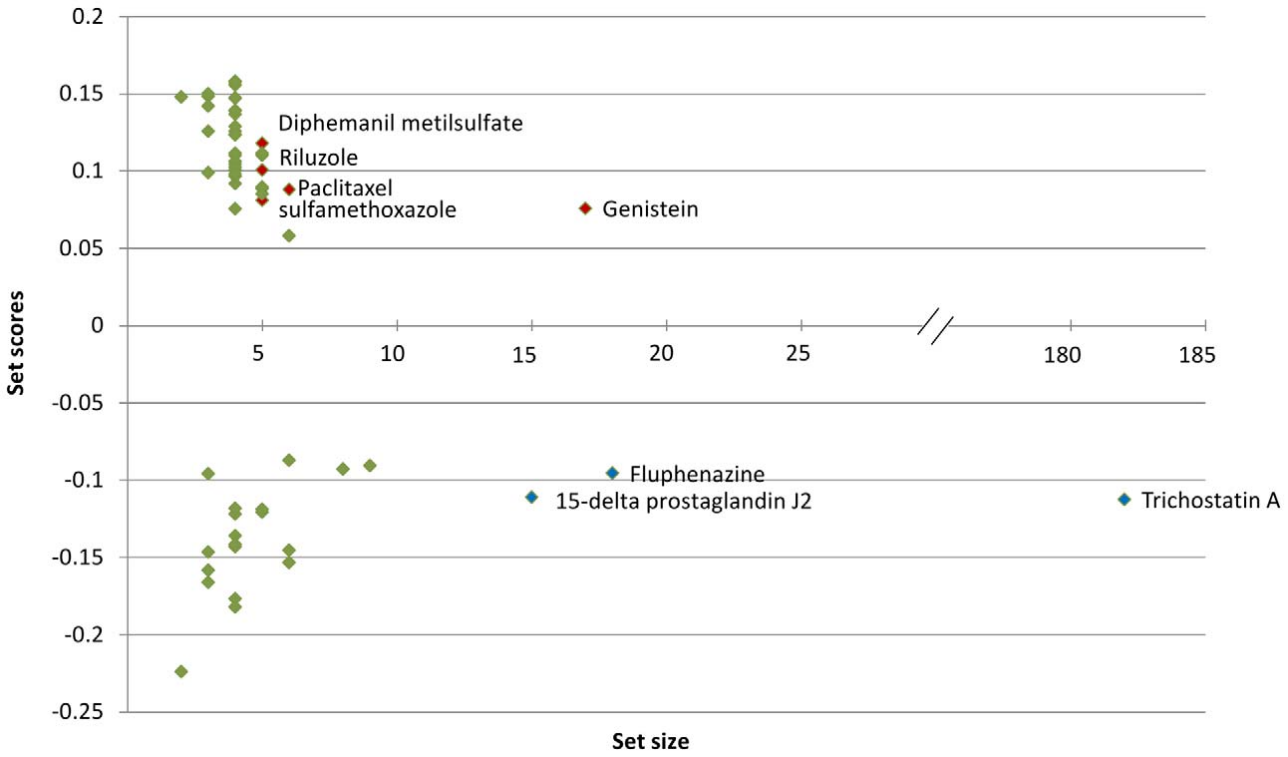
setscore-vs-setsize plot of significant connections to the cervical cancer signature. Green- significant connections; Blue- significant negative connection setscores with high PS and large setszie; Red- significant positive connection setscores with high PS and large setsize.

In the cervical cancer case study a list of 575 genes were obtained with an ENFP <0.1. Filtering the genes by requiring a minimum of log2 intensities of 6 in both groups, and an absolute log2 expression ratio of above 1, a list of 210 significant genes with p-value 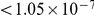 were obtained. Gene signatures were then formulated containing 210, 100, 50, 30, 20 and 10, top ranked genes, as above. These gene signatures of different lengths were then put to sscMap and the number of significant compounds examined, the gene signature of 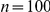 was deemed the most worthy for further scrutinising by the perturbation method, as there were only two candidate drugs for the 50-gene signature and returning no candidates for any of the shorter profiles. Perturbation signatures were generated, and together with the original one, these were put to sscMap and results analysed. The list of drugs with significant connections to the cervical cancer gene-signature were then sorted by their perturbation stability. Figure 1 shows the significant connections in relation to setscore against setsize (green) with effectors with high perturbation score and setsize highlighted other than green.

#### The connections to drugs

The cervical cancer case study returned a list of 16 candidate compounds with a PS of 1 whose expression profiles were inversely connected to the cervical cancer gene signature (see top of Table 2). The top candidates with high setsize were trichostatin A, fluphenazine and 15-delta prostaglandin J2. Trichostatin A, a histone deacetylase (HDAC) inhibitor, has been used in cancer research; it offers a novel approach to treating chemoresistant tumours by enhancing sensitivity to DNA damaging agents [29]. Fluphenazine is an anti-psychotic drug and a member of the phenothiazine group, which have been proposed as having an antiproliferative effect and may be successful as anticancer agents [30]. 15-delta prostaglandin J2 has been shown to demonstrate inhibition of cell proliferation in cancers by peroxisome proliferator-activated receptor-gamma-dependent and -independent mechanisms [31].

**Table 2:** Compounds with significant connections to the cervical cancer gene signature.

REF	TabNo	SS	PS	SetNo	SetS
trichostatin A	101	101	1	−0.1124	182
fluphenazine	101	101	1	−0.0953	18
15-delta prostaglandin J2	101	101	1	−0.1109	15
gossypol	101	101	1	−0.1532	6
pyrvinium	101	101	1	−0.1452	6
rofecoxib	101	101	1	−0.087	6
clotrimazole	101	101	1	−0.1205	5
5155877	101	101	1	−0.1218	4
5707885	101	101	1	−0.143	4
etoposide	101	101	1	−0.1358	4
puromycin	101	101	1	−0.1767	4
semustine	101	101	1	−0.182	4
thiostrepton	101	101	1	−0.1414	4
etacrynic acid	101	101	1	−0.1464	3
rottlerin	101	101	1	−0.1582	3
MS-275	101	101	1	−0.2238	2
genistein	101	97	0.9604	0.076	17
paclitaxel	101	100	0.9901	0.0881	6
sulfamethoxazole	101	101	1	0.0853	5
riluzole	101	101	1	0.1007	5
diphemanil metilsulfate	101	101	1	0.1180	5

Those at the top of the table with negative setscores are candidates that may inhibit the cervical cancer phenotype. At the bottom of the table with positive setscores are compounds that may elicit a transcriptional response similar to that of cervical cancer. REF is the (drug) name for the reference set; TabNo (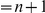) is the total number of gene signatures used in the perturbation analysis; SS is the total number of significant connections to REF from those 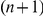 signatures; PS is the perturbation stability 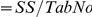, and SetNo is the setscore; SetS is the setsize.

There were 3 drugs that had positive connection scores to the cervical cancer signature with a perturbation stability of 1 and a SetS of 5 (diphemanil metilsulfate, riluzole and sulfamethoxazole) along with genistein and paclitaxel which had 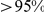 PS but a larger SetS. Diphemanil metilsulfate, riluzole and sulfamethoxazole had setscores ranging from 0.1180 to 0.0853 (top of Figure 1). Diphemanil metilsulfate is a synthetic quaternary ammonium compound [32], but little is known of its use in cancer research. Riluzole, an ion channel modulator, has been shown to demonstrate by estrogen receptor stress an inhibition of DNA synthesis and apoptosis in certain cell lines [33]. Sulfamethoxazole, an antibacterial agent, has been used for treatment of infections in patients with cancer, in combination with trimethoprim with minimal toxicity [34]. A comprehensive list of drugs connected to the Cervical cancer gene signature can be found in the supplementary Table S2.

### Letrozole treated breast cancer

The letrozole treated breast cancer study was an example where the opposite direction to previous examples was sought. Here we would be looking for compounds that have a similar effect as letrozole, with candidates in the adverse list having a role that was contrasting with letrozole. In compiling the gene signature, t-tests returned a list of 33 genes using a ENFP <3. Since this was a manageable list, and probably related to the direct effect of the treatment, no further stringency filters were applied thus removing experimenter involvement. As in the above two cases, signatures were created using 33, first 20 and first 10 of the most significant genes. They were analysed by sscMap and based on the number of drugs the gene signature of 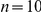 was selected for the perturbation analysis as described in the examples above. The 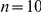 signature returned a list of 461 drugs with significant connection to it, 312 had positive connection score, 304 had perturbation stability 1. The return of 304 compounds is a reflection of the signature compiled as a result of the t-test, that would appear to have diverse effects on specific genes. As this was a large number, the list was furthered filtered by setsize. In total 30 candidate drugs with positive connection scores were retrieved satisfying PS = 1 and SetS 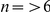 (see top of Table 3). The SetS for this list ranged from 19 to 6 with SetNos from 0.6797 to 0.2719.

**Table 3:** Compounds with significant connections to the gene signature for letrozole treatment in breast cancer.

REF	TabNo	SS	PS	SetNo	SetS
chlorpromazine	11	11	1	0.4022	19
fluphenazine	11	11	1	0.2719	18
15-delta prostaglandin J2	11	11	1	0.4478	15
nordihydroguaiaretic acid	11	11	1	0.3359	15
resveratrol	11	11	1	0.6157	9
0179445-0000	11	11	1	0.4072	8
carbamazepine	11	11	1	0.3911	8
deferoxamine	11	11	1	0.5074	8
indometacin	11	11	1	0.3732	8
methotrexate	11	11	1	0.5323	8
felodipine	11	11	1	0.4632	7
nifedipine	11	11	1	0.3734	7
0173570-0000	11	11	1	0.6153	6
0175029-0000	11	11	1	0.6159	6
beta-escin	11	11	1	0.455	6
citiolone	11	11	1	0.4647	6
cloperastine	11	11	1	0.5028	6
cotinine	11	11	1	0.6079	6
dipyridamole	11	11	1	0.6797	6
ethotoin	11	11	1	0.5104	6
eucatropine	11	11	1	0.5135	6
gossypol	11	11	1	0.3725	6
ketoprofen	11	11	1	0.3953	6
lomefloxacin	11	11	1	0.3204	6
loperamide	11	11	1	0.3829	6
meclofenoxate	11	11	1	0.3914	6
medrysone	11	11	1	0.6724	6
oxaprozin	11	11	1	0.4627	6
Prestwick-674	11	11	1	0.4868	6
tinidazole	11	11	1	0.3298	6
estradiol	11	10	0.9091	−0.1921	37
diethylstilbestrol	11	11	1	−0.5198	6
alprostadil	11	11	1	−0.4043	7
PHA-00745360	11	11	1	−0.5073	8
fludrocortisone	11	11	1	−0.5622	8
genistein	11	11	1	−0.3865	17
wortmannin	11	11	1	−0.3153	18

Those at the top of the table with positive setscores can elicit a transcriptional response similar to that of letrozole. At the bottom of the table with negative setscores are compounds that may elicit a transcriptional response opposite to that of letrozole. REF is the (drug) name for the reference set; TabNo (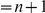) is the total number of gene signatures used in the perturbation analysis; SS is the total number of significant connections to REF from those 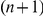 signatures; PS is the perturbation stability 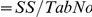, and SetNo is the setscore; SetS is the setsize.

#### The connections to drugs

**Figure 2:**
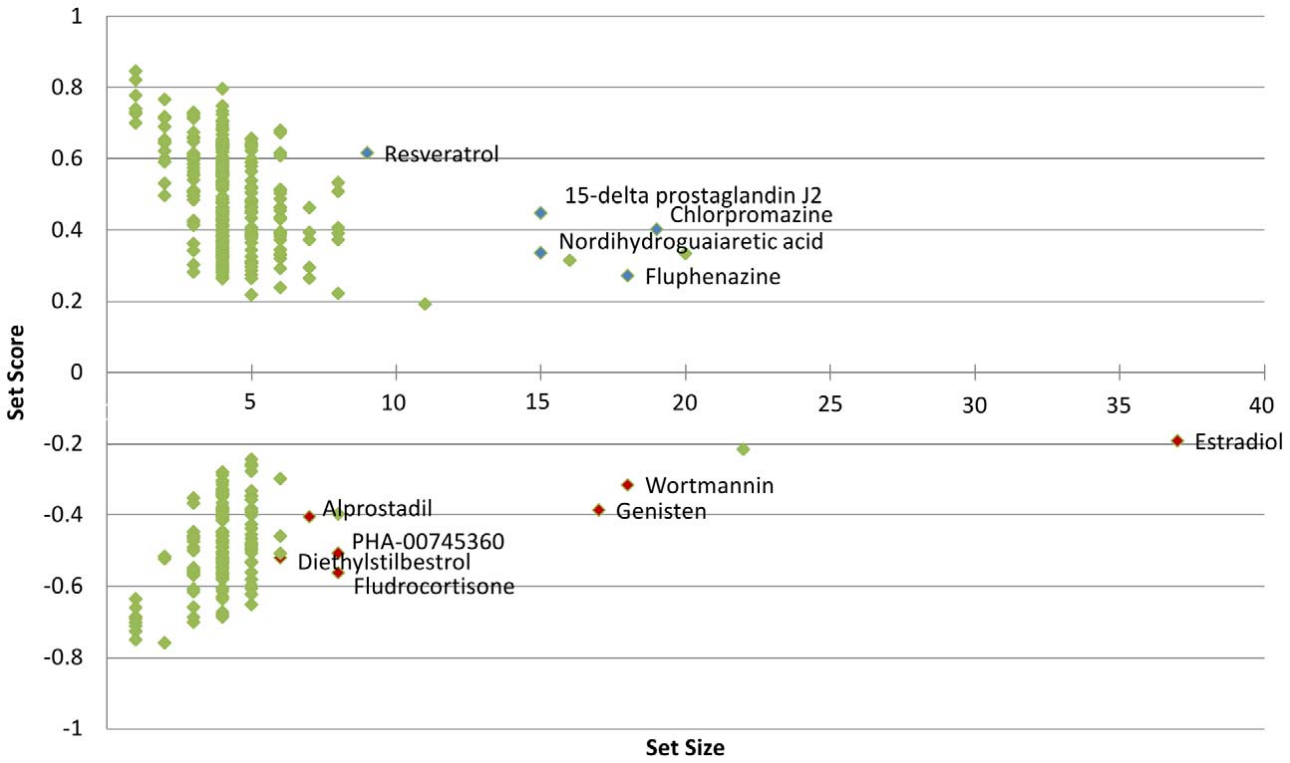
Significant connections to the letrozole treatment signature in breast cancer. Green- significant connections; Blue- significant positive connection setscores with high PS and large setsize; Red- significant negative connection setscores with high PS and large setsize.

The top-ranking compounds, shown in Figure 2, had two representative phenothiazines; chlorpromazine and fluphenazine. Chlorpromazine has shown cytoxicity towards cancerous cell lines, and there is evidence to suggest that there may be antineoplastic effects of antipsychotic drugs in general, with schizophrenia patients receiving antipsychotic medications having reduced cancer risk [35]. 15-delta prostaglandin J2 was also present along with nordihydroguaiaretic acid and resveratrol. Nordihydroguaiaretic acid, a 5-lipoxygenase inhibitor, has been shown to act as a lead compound with the ability to induce a type of apoptosis (anoikis), with no detectable toxicity [36]. Resveratrol, a naturally occurring phytoalexin present in grapes, has been studied for its therapeutic effects in cancer and its chemotherapeutic and chemopreventive abilities [37].

For opposite connections, the letrozole treated breast cancer datasets generated a list of 6 compounds where SetS was 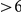 with perturbation stability of 1, (lower half of Figure 2), with estradiol included for a 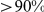 PS, being present in 10 of 11 stability tests, because of its large SetS of 37. The SetS ranged from 6 to 37 with the SetNos ranging from −0.5622 to −0.1921. The top-rated compounds were wortmanin and genistein. Wortmannin, an inhibitor of PI3K, and genistein, a phytoestrogen down-regulating tyrosine kinase, have been used in cancer research demonstrating inhibition of cancer motility and EGFR signaling inhibition, respectively [38], [39]. A comprehensive list of drugs connected to the Letrozole-treated breast cancer gene signature can be found in the supplementary Table S3.

## Discussion

### Acute Myeloid Leukemia

AML is a heterogenous disease, where targeted therapies have arisen to look at histone deacetylase inhibitors, demethylating agents, farnesyltransferase and FLT-3 inhibitors, but where the first targeted therapy is all-*trans*-retinoic acid (ATRA) [40]. The results presented in Table 1 supplied two candidates for which information is quite minimal in terms of cancer treatment, 5186223 and Prestwick-691, so interpretation of their usefulness as therapeutics is restrained and poses interesting laboratory experiments and a reminder of the information that may not be currently available in drug discovery.

TTNPB is a retnoic acid that binds to the retinoic acid receptor (RAR), which is an appealing connection given the amount literature on ATRA for treatment of an AML subtype acute promyelocytic leukemia [41], [42]. It has been reported that AML2 and AML1 expression can be induced by retinoic acid with ATRA having reduced effect on non-M3 AML (any type other than acute promyelocytic leukemia) as it acts on the RAR pathway[43], [44]. Despite this retinoid differentiation therapy has shown promising results *in vitro* [45]. Recently, Tsai *et al.* have suggested that a high affinity retinoic X receptor (RXR) may be a candidate for non-M3 AML treatment, stating also that this is possible through co-stimulation of both RAR and RXR receptors to be involved in the differentiation of non-M3 AML [46]. It was interesting that iloprost was present. Iloprost has been shown to inhibit tumour formation by the role of activation of PPAR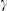 nuclear receptors [47]. It also has an inhibitory effect on the Transforming Growth Factor-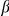 (TGF-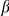), a cytokine which promotes differentiation of Th17 cells. Th17 cells have been said to progress or aid in development of AML [48]. This connection was a demonstration of the multiple pathways and diversity of targeted therapeutics and an advocate for laboratory research in understanding the most beneficial treatments for individuals. Co-dergocrine mesilate as a candidate therapeutic was an interesting finding as being an inhibitor of norepinephrine it may play a role in the decrease of cancer proliferation by stimulating presynaptic dopamine receptors. It has also a role in its activation having an effect on gene expression in estrogen-stimulated TGF-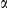 [49].

### Cervical cancer

The cervical cancer results showed by way of the top ranked candidates in the adverse list that estrogen is a factor in candidate selection, and it has been shown that estrogen receptor alpha (ER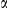) is an important instigator in cancer [50]. This would explain the presence of the estrogen related pathways in the adverse list (top half of Figure 1), with riluzole and genisten cited. Diphemanil metilsulfate has no apparent information on its effects in cancer and Sulfamethoxazole being used as an antibacterial therapeutic. The sscMap's ability to select these candidates demonstrates its heightened sensitivity to the significant list of genes analysed. The fact that the top ranked adverse candidates are either estrogen related or impervious to cervical cancer is evidence that they may be ineffective in altering the phenotype of the disease favourably.

The top therapeutic candidates in Figure 1 (lower half) being trichostatin A, fluphenazine and 15-delta prostaglandin J2 were regarded as good hits with trichostatin A (TSA) already being used in studies to treat cervical cancer in combination with bortezomib and seen to retard tumour growth [51]. Fluphenazine as a member of the phenothiazine family has been used as an antipsychotic drug but studies have shown that they have an ability to induce apoptosis and demonstrate anti-tumour activity *in vivo* [30]. 15-delta prostaglandin J2 has been demonstrated to induce growth arrest or inhibition in several tumors and cancer cell lines including breast and colon through an activation of PPAR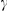 [31], [52]. That these three top hits have differentiating applications demonstrates the genetic variability in cervical cancers. Recent treatments have tended towards epidermal growth factor receptor (EGFR) and vascular endothelial growth factor VEGF signalling pathways with therapeutics focusing on adenosine triphosphate inhibitors and monoclonal antibodies [53]. All three candidates have been shown in studies to have an effect on the EGFR pathway with TSA having been shown to enhance sensitivity of TRAIL-resistant ovarian cancer cells by inhibition of the EGFR pathway [54]. Fluphenazine is a calmodulin inhibitor, with calmodulin mediating the elevations of intracellular Ca2+ activating EGFR-dependent signalling [55]. This is an indication that the gene signature analysed by the sscMap retrieved selective therapeutics by virtue of connections between strong candidates. The 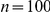 signature was a quite long list of pertinent genes, which could have led to variable results. This is an important aspect of the perturbation approach, which can remove ‘weaker’ candidates by placing them further down the list which acts as an indicator of their resolve. The candidates represented in the bottom half of Table 3 suggests a similar transcription response to the disease. They had strong attributes in PS and SetSize, therefore suggesting of their inability to benefit the disease phenotype. As stated above, the fact that ER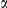 is an important instigator supports the candidates in the bottom half of Table 3 (estrogen related or impervious to cervical cancer).

### Letrozole treated Breast cancer

As letrozole, an aromatase inhibitor, offers a reduction of estrogen production it would seem likely that the drugs that would be present in the adverse list (Figure 2 lower half), would be compounds related to estrogen and estrogen receptor targets. This is the case with the selection of wortmannin, an inhibitor of PI3k, being shown to have no effect on estrogen deprived cell lines [22]. Wortmannin in that particular study was used to inhibit the PI3K/AKT pathway to restore cell sensitivity to tamoxifen (an antiestrogen) to show the interaction between the ER and growth factor receptor pathways. Also present were estradiol, genistein (phytoestrogen) and diethylstilbestrol (a synthetic estrogen) which were indicators of possible adverse effects as all these compounds would contrast the effect being afforded by letrozole therapy. Diethylstilbestrol was a drug administered to women during pregnancy to prevent miscarriage (from 1940s to 1960s) that now show higher increased rates of breast cancer. Their daughters, now at an age for diagnosis, also have an increased incidence of breast cancer, with a rate ratio of 2.5 compared to non-diethylstilbestrol-exposed [56]–[58].

The top therapeutic candidates that were supplied in Figure 2 (top half), (chlorpromazine, fluphenazine, 15-delta prostaglandin J2, nordihydroguaiaretic acid and resveratrol) are all strong treatments in cancer research. The phenothiazines (chlorpromazine and fluphenazine) are drugs used to treat bipolar and psychotic disorders, with chlorpromazine having been demonstrated to have moderate inhibitory effects on BCRP (the breast cancer resistance protein), thus reducing its multidrug resistance effects [59]. Resveratrol has many applications in cancer research, as it inhibits cellular events linked with initiation, promotion and progression [60]. 15-delta prostaglandin J2 are emerging as potent antitumour agents, being shown to have growth inhibitory effects and apoptotic capabilities [61]. Nordihydroguaiaretic acid, Resveratrol and 15-delta prostaglandin J2 are therapeutics involved in prostaglandin inhibition [61]–[63], in turn prostaglandin is a potent inducer of aromatase expression, also induces Ciclooxygenase-2 (COX-2) expression [64], and has a role in carcinogenesis. This pathway is also exploited by letrozole which has been shown to block the aromatase up-regulated by prostaglandin [65], and there would be an obvious connection between these therapeutics.

The sscMap's proposed candidates are either therapeutics that have a strong involvement in cancer treatment or may act in a similar pathway as letrozole suggesting that the gene signature, although containing only 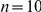 genes (probesets), could accurately summate the pertinent expression levels in the dataset. The list of compounds in Figure 2 demonstrate the utility of the enhanced significance selection process offered by the gene-signature perturbation method.

### The strength of the perturbation approach

The strength of the perturbation approach in its ability to rank therapeutics can be gauged by contrasting Table 2 and Table 4 of the cervical cancer case study, with Table 4 ranking candidates without implementing the perturbation stability measure. Without the ability to achieve a manageable list length it creates an issue of where to decide to stop looking for candidates, with Table 4 being thresholded at SetS greater than 4. Of course we would expect the top couple of candidates to be present in both tables as having a large SetS and SetNo would be indicator that the original sscMap was able to retrieve strong candidates. Here we can see trichostatin A, fluphenazine and 15-delta prostaglandin J2 have performed well in both Tables. Perturbation has the ability to filter the candidates already represented by their robustness and create an advanced system of therapeutic selection. The top halves of Table 4 and Table 2 representing the contrasting transcription profiles, after the top 3, differ by their ability to withstand systematic checks. This increases the confidence in the list attained and confers more reliability in the experimenters ability to retrieve viable candidates. The original ranking attributes, although successful, fail to convey any increased ability of one candidate over another. The perturbation enhancement delivers in the ability to derive if this is a strong candidate, and can be utilised in tandem with these other attributes, as is the case with genistein, where in Table 4 it is the top candidate in the similar transcription profile to the disease phenotype(and we would have expected it to be there) but this is not the case when we check for robustness where it moves further down the ranking. Thus it implies that checking for robustness can be a deciding factor in the ability to decide therapeutic selections, reduce list sizes and increase the potential of altering the phenotype.

**Table 4:** Without using the perturbation analysis this table (thresholded at SetS≥5) lists compounds with significant connections to the cervical cancer gene signature.

REF	SetS	GeneLen	SetNo
trichostatin A	182	100	−0.1124
fluphenazine	18	100	−0.0953
15-delta prostaglandin J2	15	100	−0.1109
resveratrol	9	100	−0.0904
0179445-0000	8	100	−0.0927
gossypol	6	100	−0.1532
pyrvinium	6	100	−0.1452
rofecoxib	6	100	−0.0870
clotrimazole	5	100	−0.1205
halcinonide	5	100	−0.1188
trimethoprim	5	100	0.0812
sulfamethoxazole	5	100	0.0853
riluzole	5	100	0.1007
naphazoline	5	100	0.0888
iproniazid	5	100	0.1117
hydrochlorothiazide	5	100	0.0898
guanadrel	5	100	0.1103
diphemanil metilsulfate	5	100	0.1180
prilocaine	6	100	0.0582
paclitaxel	6	100	0.0881
genistein	17	100	0.0760

REF is the (drug) name for the reference set; SetS is the setsize; GeneLen is the length of gene signature; SetNo is the setscore.

### Conclusions

Differential gene expression offers a viable window into the complexity of the cellular response to deviation from normal activity and to drug treatment. Gene-expression connectivity mapping is an important technological development for the establishment and interpretation of connections among genes, drugs, and diseases. The gene-signature perturbation approach is a further advance towards robust and effective connectivity mapping. It was conceived in an attempt to gauge the stability of an identified compound's connection to gene signatures. This extended approach is favourable over the existing technique, which lists the results by either the setscore or setsize, in assessing the therapeutics influence on the compiled signature. This offers insight into pertinent genes as well as therapeutic selection. It removes experimenter influence in assessing therapeutic applicative assumptions by unveiling their accountability to perturbation. The merits of using the sscMap software have been previously addressed [5], [6], this now coupled with an unbiased method of therapeutic selection enhances its utility.

The case studies we have analysed here returned favorable results and insightful leads. For the letrozole treated breast cancer case, we could see an obvious ‘trend’ occurring in the results with adverse compounds being those of an estrogen derived pathway, in contrast to the favourable candidates with hypothesised similarity in effect. These were comparable by the mode of action with the pathway being prostaglandin inhibition which was a recurrent theme in the top hits retrieved. The results of the AML case study supplied interesting candidates with, among others, a potent retinoic acid TTNPB. The fact that ATRAs are used as therapeutics for AML suggests this is a very interesting connection list with very little known about the two candidates ranked above (5186223 and Prestwick-691), which offers an intrigue to their potential use. The cervical cancer results for the top ranked candidate compounds revealed a strong association with the EGFR pathway. These results demonstrate the ability of the perturbation method to make incisive interpretations achieved with varying sizes of gene signatures ranging from 10 in the Letrozole study, to 100 in the cervical cancer study.

The perturbation approach is a conceptually simple but practically useful idea; it added real value to the connectivity mapping process allowing for increased specificity in the identification of possible therapeutic candidates. The ‘strength’ of each candidate compound derived from checking for robustness against perturbation offers a viable unbiased method of candidate selection for further experimentation. Embracing such criteria is an important aspect of enhancing research in drug discovery establishing potential uses for existing drugs in diseases that have poor prognosis. Future direction will likely lend itself to the understanding of ideal gene signature size to reduce user selectivity and offer a more pragmatic approach. It is very much needed now in connectivity mapping to develop a systematic and coherent procedure or protocol that enables biomedical researchers to construct high quality gene signatures and determine their optimal lengths automatically. One possible strategy to achieve this, when the number of differentially expressed genes is large from microarrays, is to try to incorporate other biological knowledge in constructing gene signatures, for example, by requiring the genes included in a signature belonging to a small number of dominate pathways or biological processes. This will entail sophisticated computational procedures involving the retrieving and compiling of existing biological knowledge from external databases such as GO, KEGG PATHWAY, and NCI-Nature PID. We see future research in this direction to be greatly beneficial to gene-expression connectivity mapping and its wide applications.

## Methods

### The Perturbation approach

In this section we describe the procedures of the perturbation approach to connectivity mapping. A gene-signature, as described in previous studies [3], [5], is a concise list (threshold applied by a mathematical variable) of statistically differentiated genes with their regulation status (up or down) specified, which collectively characterise the biological state of a researcher's interest. A gene-signature is usually the result of some gene-expression profiling (e.g. DNA microarray) experiments investigating a particular biological condition, with the most significantly differentially expressed genes selected. A gene-signature should capture the most prominent feature(s) of the biological state it represents, while a reference gene-expression profile, in the context of connectivity mapping, represented in non-parametric fashion, is intended to provide a more comprehensive description of the reference state. This reference gene-expression profile contains rank-ordered genes of a profile compared to a vehicle treated control. There are currently 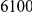 individual reference gene-expression profiles in the sscMap core-database, each representing a biological state induced by treating cells with a compound as compared to vehicle control. In total 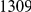 compounds were represented with 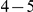 microarray hybridisations per compound on average (the actual number of hybridisations with each compound varies). In this study, all individual reference profiles with the same compound are designated as belonging to *a reference set*, and SetSize refers to the number of microarray hybridisations with that compound. Hence a set of reference profiles with the same compound (a reference set) collectively characterise the effects of that compound.

#### Connecting a gene signature to reference sets

For a given gene-signature, whether it is the original signature, or a perturbation signature (see below for detailed definition) derived from the original one, we calculate a connection score (setscore) between the signature and each set of reference profiles, and also calculate the associated p-value using the method described in [5]. If the p-value is lower than a pre-set threshold, the connection is declared as statistically significant. In our connectivity mapping exercise we always set the threshold p-value as 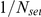, where 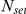 is the total number of reference sets being queried. By setting the threshold p-value as such, we expect to have one false connection per gene-signature. A false connection means that whereas in reality there is no connection between the gene-signature and the reference set, the p-value happens to be lower than the threshold purely by chance.

#### Generating perturbation signatures

Suppose 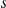 is a gene-signature of length 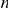, where 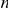 is the number of genes (probesets) in the signature. We can generated 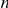 perturbation gene-signatures of length 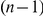 by taking one gene out each time, with replacement. We shall use 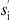 to denote a perturbation signature in which the 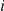
th gene in the original signature 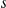 is excluded. The symbol 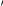 indicates that this is a perturbation signature.

#### Perturbation stability

For each of the 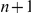 gene-signatures (one original plus 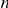 perturbation signatures), we use sscMap to establish the connections between the signature and the 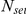 reference sets, and use the criteria described above to determine whether the connections are statistically significant or not. For each reference set 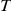, its connections to the original gene signature 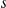 and the 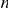 perturbation gene-signatures 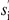
s are examined. If the connection between signature 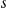 and the reference set 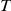 is statistically significant, then we examine whether 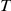 also has significant connections to the 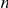 perturbation signatures. We define “perturbation stability” as the proportion of significant connections out of the total number 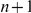. So a perturbation stability 1 means that not only the reference set has a significant connection to the original signature 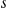, but also the reference set has significant connections to all the perturbation signatures 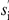
s derived from the original one. Our rationale of introducing the perturbation stability is to test whether a discovered connection is robust and stable enough against small changes in the signature (perturbations). An analogy to this exercise in the physical world is that to test whether a physical object stands stable, you can give it some small pushes from all different directions to see if it still stands. The perturbation stability gives us an effective measure to analyse the sometimes large number of significant connections in the connectivity mapping exercise. It allows us to concentrate on those connections that are most stable and thus increases the rate of success in following-up investigations.

#### Data collection, processing, and analysis

The zipped files for each of the three case studies were downloaded from GEO website was extracted to reveal the array data file. The R package software of Affymetrix Micro Array Suite 5.0, MAS5, was used to extract the expression data for each of the arrays in the three studies. Each of the individual studies arrays were separated into control and effector groups with their relevant expression incorporated into a single file for statistical analysis. As with the Lamb *et al*. analysis, normalisation was not incorporated. The gene signature was formulated by compiling the samples gene-expression levels, separated into control/treatment and the formulation of statistical difference by t-test. Ranking these genes by the t-test results supplied a list of significant genes determined by the expected number of false positives. All gene signatures were then analysed by sscMap, which has a built-in core database for over 6000 gene expression profiles. A prerequisite is the use of (or conversion to) Affymetrix HG-U133A probe-set IDs.

The sscMap reads gene signature files in tab delimited format with a column containing the Affymetrix IDs and also a column for indication of increased or decreased expression (+1 or −1). Once executed an output file is generated that includes candidate compounds with significant connections to the signature. Given a particular gene-signature of interest, a list of all possible perturbation signatures are derived. The connectivity mapping is then carried out on all the perturbation signatures and the original one, and a data matrix of the connections' significance index can be compiled, by the summation of the therapeutic instances per list, thus incorporating all the results. Each connection's ‘strength’ is based on its ability to remain unperturbed by the absence of a single gene, which is quantified by the perturbation stability described in the previous section.

## Supporting Information

Table S1 **A comprehensive list of drugs connected to the AML gene signature.** (a) Of the five therapeutics retrieved by the sscMap, listed below, only one had a stable perturbation score of 1. (b) The remaining 25 therapeutics had positive setscores, and were deemed to be adverse candidates. Of these 14 had a perturbation score of 1, thus indicating that they were present throughout the perturbation assessment.
(DOC)

Table S2 **A comprehensive list of drugs connected to the Cervical Cancer gene signature.** (a) Therapeutic candidates. The therapeutics returned varied from 182 to 2 in setsize with 16 returning a perturbation score of 1. For the analysis only the top three candidates were looked at in detail. (b) Adverse drug candidates returned from the sscMap-perturbation study. The setsizes varied from 17 to 2 with 26 perturbation scores of 1. For the analysis we wanted to look at a selected few so only candidates over a setsize of 5 were analysed, for this we included Paclitaxel and Genistein (in Bold), as the Perturbation scores were appreciably high coupled with the high setsize.
(DOC)

Table S3 **A comprehensive list of drugs connected to the Letrozole-treated breast cancer gene signature.** (a) The therapeutics list when filtered (setsize with n>6) generated 30 candidates ranging in setscore from 6 to 19. (b) Candidates were also selected that would have an opposite to that of letrozole on breast cancer. These were the negative setscore'd compounds. With the filter applied this generated 12 candidates. This list contained 6 candidates with a perturbation score of 1.
(DOC)
