# Supplementary material for: Identification of Candidate Small-Molecule Therapeutics to Cancer by Gene-Signature Perturbation in Connectivity Mapping
Source: PLoS One. 2011 Jan 31;6(1):e16382. doi: 10.1371/journal.pone.0016382 (PMC3031567; doi:10.1371/journal.pone.0016382)
Supplement: Table S1 — A comprehensive list of drugs connected to the AML gene signature. (a) Of the five therapeutics retrieved by the sscMap, listed below, only one had a stable perturbation score of 1. (b) The remaining 25 therapeutics had positive setscores, and were deemed to be adverse candidates. Of these 14 had a perturbation score of 1, thus indicating that they were present throughout the perturbation assessment. (DOC) [file pone.0016382.s001.doc]

## Acute Myeloid Leukaemia results

**A gene list of 30 was chosen, after critical assessment of various gene signature lengths by sscMap, as a candidate for perturbation connections. The list had five negative setscores in a significant list of 30 candidates. An n+1 perturbation assessment giving 31 lists with which the therapeutics had to be checked against.**

**Table S1 (a) Of the five therapeutics retrieved by the sscMap, listed below, only one had a stable perturbation score of 1.**

| **REFSETNAME** | **Tables Count** | **Sum-Sigs** | **perturb stabil** | **SetScore** | **SetSize** |
| --- | --- | --- | --- | --- | --- |
| 5186223 | 31 | 31 | 1 | -0.38792914 | 1 |
| Prestwick-691 | 31 | 28 | 0.903225806 | -0.34377558 | 3 |
| TTNPB | 31 | 17 | 0.548387097 | -0.30490453 | 2 |
| co-dergocrine mesilate | 31 | 15 | 0.483870968 | -0.23778468 | 4 |
| Iloprost | 31 | 12 | 0.387096774 | -0.27963017 | 3 |

**Refsetname:** the therapeutic candidate

**Tables Count**: the sum of the lists

**Sum-Sigs**: is the additive presence of the therapeutic in the lists

**Perturb stabil**: the perturbation stability score generated by the division of Tables Count by Sum-Sigs

**Table S1 (b) The remaining 25 therapeutics had positive setscores, and were deemed to be adverse candidates. Of these 14 had a perturbation score of 1, thus indicating that they were present throughout the perturbation assessment.**

| **REFSETNAME** | **Tables Count** | **Sum-Sigs** | **perturb stabil** | **SetScore** | **SetSize** |
| --- | --- | --- | --- | --- | --- |
| Solasodine | 31 | 31 | 1 | 0.15511298 | 6 |
| Dilazep | 31 | 31 | 1 | 0.19224914 | 5 |
| Neomycin | 31 | 31 | 1 | 0.17056859 | 5 |
| Tranylcypromine | 31 | 31 | 1 | 0.25885773 | 5 |
| Carbarsone | 31 | 31 | 1 | 0.18140609 | 4 |
| Flucytosine | 31 | 31 | 1 | 0.25332607 | 4 |
| Methanthelinium bromide | 31 | 31 | 1 | 0.15994042 | 4 |
| Prestwick-1085 | 31 | 31 | 1 | 0.19945812 | 4 |
| Saquinavir | 31 | 31 | 1 | 0.20136478 | 4 |
| Seneciphylline | 31 | 31 | 1 | 0.18246289 | 4 |
| Aminocaproic acid | 31 | 31 | 1 | 0.25562765 | 3 |
| Bromperidol | 31 | 31 | 1 | 0.20698146 | 3 |
| Doxorubicin | 31 | 31 | 1 | 0.29899783 | 3 |
| Acetylsalicylic acid | 31 | 30 | 0.96774194 | 0.11397116 | 13 |
| 0179445-0000 | 31 | 30 | 0.96774194 | 0.17369696 | 8 |
| H-7 | 31 | 30 | 0.96774194 | 0.30663418 | 4 |
| Delsoline | 31 | 27 | 0.870967742 | 0.21413955 | 4 |
| LM-1685 | 31 | 24 | 0.77419355 | 0.30461114 | 3 |
| Camptothecin | 31 | 23 | 0.74193548 | 0.290754 | 3 |
| Trimethylcolchicinic acid | 31 | 20 | 0.64516129 | 0.19095957 | 4 |
| Clemizole | 31 | 19 | 0.61290323 | 0.207491 | 5 |
| Prestwick-685 | 31 | 15 | 0.48387097 | 0.20738562 | 5 |
| Oxetacaine | 31 | 12 | 0.38709677 | 0.21361684 | 5 |
| Nordihydroguaiaretic acid | 31 | 10 | 0.32258065 | 0.11379083 | 15 |
| N-phenylanthranilic acid | 31 | 5 | 0.16129032 | 0.34696394 | 1 |
