# Supplementary material for: Identification of Candidate Small-Molecule Therapeutics to Cancer by Gene-Signature Perturbation in Connectivity Mapping
Source: PLoS One. 2011 Jan 31;6(1):e16382. doi: 10.1371/journal.pone.0016382 (PMC3031567; doi:10.1371/journal.pone.0016382)
Supplement: Table S2 — A comprehensive list of drugs connected to the Cervical Cancer gene signature. (a) Therapeutic candidates. The therapeutics returned varied from 182 to 2 in setsize with 16 returning a perturbation score of 1. For the analysis only the top three candidates were looked at in detail. (b) Adverse drug candidates returned from the sscMap-perturbation study. The setsizes varied from 17 to 2 with 26 perturbation scores of 1. For the analysis we wanted to look at a selected few so only candidates over a setsize of 5 were analysed, for this we included Paclitaxel and Genistein (in Bold), as the Perturbation scores were appreciably high coupled with the high setsize. (DOC) [file pone.0016382.s002.doc]

## Cervical cancer results

**A significant list containing 100 genes were put to the sscMap for perturbation analysis based on the performance of the list for significant connections. After assessment it returned 61 connections with 22 negative setscores and 39 positive setscores. All are listed below.**

**Table S2 (a) Therapeutic candidates. The therapeutics returned varied from 182 to 2 in setsize with 16 returning a perturbation score of 1. For the analysis only the top three candidates were looked at in detail.**

| **REFSETNAME** | **Tables Count** | **Sum-Sigs** | **perturb stabil** | **SetScore** | **SetSize** |
| --- | --- | --- | --- | --- | --- |
| trichostatin A | 101 | 101 | 1 | -0.11242253 | 182 |
| fluphenazine | 101 | 101 | 1 | -0.09533414 | 18 |
| 15-delta prostaglandin J2 | 101 | 101 | 1 | -0.11091284 | 15 |
| gossypol | 101 | 101 | 1 | -0.1531598 | 6 |
| pyrvinium | 101 | 101 | 1 | -0.14523452 | 6 |
| rofecoxib | 101 | 101 | 1 | -0.08697671 | 6 |
| clotrimazole | 101 | 101 | 1 | -0.12051814 | 5 |
| 5155877 | 101 | 101 | 1 | -0.1217649 | 4 |
| 5707885 | 101 | 101 | 1 | -0.14295163 | 4 |
| etoposide | 101 | 101 | 1 | -0.13583039 | 4 |
| puromycin | 101 | 101 | 1 | -0.17666 | 4 |
| semustine | 101 | 101 | 1 | -0.18199181 | 4 |
| thiostrepton | 101 | 101 | 1 | -0.14143623 | 4 |
| etacrynic acid | 101 | 101 | 1 | -0.14638301 | 3 |
| rottlerin | 101 | 101 | 1 | -0.15817108 | 3 |
| MS-275 | 101 | 101 | 1 | -0.22384015 | 2 |
| 0179445-0000 | 101 | 94 | 0.930693069 | -0.09268733 | 8 |
| Pimozide | 101 | 93 | 0.920792079 | -0.11808881 | 4 |
| prednicarbate | 101 | 91 | 0.900990099 | -0.09569554 | 3 |
| Resveratrol | 101 | 87 | 0.861386139 | -0.09042511 | 9 |
| 0297417-0002B | 101 | 80 | 0.792079208 | -0.16600805 | 3 |
| Halcinonide | 101 | 37 | 0.366336634 | -0.11876412 | 5 |

**Refsetname:** the therapeutic candidate

**Tables Count**: the sum of the lists

**Sum-Sigs**: is the additive presence of the therapeutic in the lists

**Perturb stabil**: the perturbation stability score generated by the division of Tables Count by Sum-Sigs

**Table S2 (b) Adverse drug candidates returned from the sscMap-perturbation study. The setsizes varied from 17 to 2 with 26 perturbation scores of 1. For the analysis we wanted to look at a selected few so only candidates over a setsize of 5 were analysed, for this we included Paclitaxel and Genistein (in Bold), as the Perturbation scores were appreciably high coupled with the high setsize.**

| **REFSETNAME** | **Tables Count** | **Sum-Sigs** | **perturb stabil** | **SetScore** | **SetSize** |
| --- | --- | --- | --- | --- | --- |
| diphemanil metilsulfate | 101 | 101 | 1 | 0.118028 | 5 |
| Riluzole | 101 | 101 | 1 | 0.100692 | 5 |
| Sulfamethoxazole | 101 | 101 | 1 | 0.085336 | 5 |
| Dobutamine | 101 | 101 | 1 | 0.158309 | 4 |
| Suxibuzone | 101 | 101 | 1 | 0.157677 | 4 |
| Acemetacin | 101 | 101 | 1 | 0.15579 | 4 |
| Terazosin | 101 | 101 | 1 | 0.147554 | 4 |
| Cloxacillin | 101 | 101 | 1 | 0.147256 | 4 |
| Asiaticoside | 101 | 101 | 1 | 0.1394 | 4 |
| Dipivefrine | 101 | 101 | 1 | 0.136933 | 4 |
| Harmol | 101 | 101 | 1 | 0.128983 | 4 |
| Glafenine | 101 | 101 | 1 | 0.125784 | 4 |
| Pergolide | 101 | 101 | 1 | 0.12366 | 4 |
| Disopyramide | 101 | 101 | 1 | 0.123534 | 4 |
| Prestwick-857 | 101 | 101 | 1 | 0.111607 | 4 |
| Amikacin | 101 | 101 | 1 | 0.110251 | 4 |
| Loracarbef | 101 | 101 | 1 | 0.106385 | 4 |
| Carbenoxolone | 101 | 101 | 1 | 0.104734 | 4 |
| Nizatidine | 101 | 101 | 1 | 0.102559 | 4 |
| Scoulerine | 101 | 101 | 1 | 0.098292 | 4 |
| Altretamine | 101 | 101 | 1 | 0.092007 | 4 |
| Trapidil | 101 | 101 | 1 | 0.15017 | 3 |
| Alfadolone | 101 | 101 | 1 | 0.148564 | 3 |
| Penbutolol | 101 | 101 | 1 | 0.142209 | 3 |
| Thiamine | 101 | 101 | 1 | 0.125911 | 3 |
| 5252917 | 101 | 101 | 1 | 0.1481 | 2 |
| **Paclitaxel** | **101** | **100** | **0.990099** | **0.088076** | **6** |
| Carteolol | 101 | 97 | 0.96039604 | 0.100853 | 4 |
| **Genistein** | **101** | **97** | **0.960396** | **0.075956** | **17** |
| Vinburnine | 101 | 96 | 0.95049505 | 0.139308 | 4 |
| Guanadrel | 101 | 92 | 0.910891089 | 0.110344 | 5 |
| Hydrochlorothiazide | 101 | 82 | 0.811881188 | 0.089751 | 5 |
| Trimethoprim | 101 | 75 | 0.742574257 | 0.081185 | 5 |
| Prilocaine | 101 | 68 | 0.673267 | 0.058229 | 6 |
| Lansoprazole | 101 | 45 | 0.445544554 | 0.096801 | 4 |
| Triprolidine | 101 | 40 | 0.396039604 | 0.075708 | 4 |
| Tetracaine | 101 | 35 | 0.346534653 | 0.099093 | 3 |
| Iproniazid | 101 | 28 | 0.277227723 | 0.111667 | 5 |
| Naphazoline | 101 | 28 | 0.277227723 | 0.088764 | 5 |
