# Supplementary material for: Identification of Candidate Small-Molecule Therapeutics to Cancer by Gene-Signature Perturbation in Connectivity Mapping
Source: PLoS One. 2011 Jan 31;6(1):e16382. doi: 10.1371/journal.pone.0016382 (PMC3031567; doi:10.1371/journal.pone.0016382)
Supplement: Table S3 — A comprehensive list of drugs connected to the Letrozole-treated breast cancer gene signature. (a) The therapeutics list when filtered (setsize with n>6) generated 30 candidates ranging in setscore from 6 to 19. (b) Candidates were also selected that would have an opposite to that of letrozole on breast cancer. These were the negative setscore'd compounds. With the filter applied this generated 12 candidates. This list contained 6 candidates with a perturbation score of 1. (DOC) [file pone.0016382.s003.doc]

## Breast cancer treated with Letrozole results

**A gene list comprising of 10 significant candidates were chosen with due diligence for perturbation connections. It generated 312 positive setscores in a significant list of 461 candidates. The perturbation list gave 304 candidates of perturbation stability equalling 1. The list was filtered by setsize with n=>6, leaving 42 candidates for assessment.**

**Table S3 (a) The therapeutics list when filtered (setsize with n=>6) generated 30 candidates ranging in setscore from 6 to 19.**

| **REFSETNAME** | **Tables count** | **Sum-Sigs** | **perturb stabil** | **SetScore** | **SetSize** |
| --- | --- | --- | --- | --- | --- |
| Chlorpromazine | 11 | 11 | 1 | 0.40223841 | 19 |
| Fluphenazine | 11 | 11 | 1 | 0.27187099 | 18 |
| 15-delta prostaglandin J2 | 11 | 11 | 1 | 0.44780244 | 15 |
| Nordihydroguaiaretic acid | 11 | 11 | 1 | 0.33592507 | 15 |
| Resveratrol | 11 | 11 | 1 | 0.61573366 | 9 |
| 0179445-0000 | 11 | 11 | 1 | 0.4071936 | 8 |
| Carbamazepine | 11 | 11 | 1 | 0.39105977 | 8 |
| Deferoxamine | 11 | 11 | 1 | 0.50739951 | 8 |
| Indometacin | 11 | 11 | 1 | 0.37324214 | 8 |
| Methotrexate | 11 | 11 | 1 | 0.53225026 | 8 |
| Felodipine | 11 | 11 | 1 | 0.46323393 | 7 |
| Nifedipine | 11 | 11 | 1 | 0.37340485 | 7 |
| 0173570-0000 | 11 | 11 | 1 | 0.61527856 | 6 |
| 0175029-0000 | 11 | 11 | 1 | 0.61587255 | 6 |
| Beta-escin | 11 | 11 | 1 | 0.45497677 | 6 |
| Citiolone | 11 | 11 | 1 | 0.46466848 | 6 |
| Cloperastine | 11 | 11 | 1 | 0.50277622 | 6 |
| Cotinine | 11 | 11 | 1 | 0.60794264 | 6 |
| Dipyridamole | 11 | 11 | 1 | 0.67970764 | 6 |
| Ethotoin | 11 | 11 | 1 | 0.51039717 | 6 |
| Eucatropine | 11 | 11 | 1 | 0.5134846 | 6 |
| Gossypol | 11 | 11 | 1 | 0.37252882 | 6 |
| Ketoprofen | 11 | 11 | 1 | 0.3953019 | 6 |
| Lomefloxacin | 11 | 11 | 1 | 0.32035819 | 6 |
| Loperamide | 11 | 11 | 1 | 0.38286913 | 6 |
| Meclofenoxate | 11 | 11 | 1 | 0.39140128 | 6 |
| Medrysone | 11 | 11 | 1 | 0.67239566 | 6 |
| Oxaprozin | 11 | 11 | 1 | 0.46265383 | 6 |
| Prestwick-674 | 11 | 11 | 1 | 0.48682736 | 6 |
| Tinidazole | 11 | 11 | 1 | 0.32975739 | 6 |
| Other data omitted.... |  |  |  |  |  |

**Refsetname:** the therapeutic candidate

**Tables Count**: the sum of the lists

**Sum-Sigs**: is the additive presence of the therapeutic in the lists

**Perturb stabil**: the perturbation stability score generated by the division of Tables Count by Sum-Sigs

**Table S3 (b) Candidates were also selected that would have an opposite to that of letrozole on breast cancer. These were the negative setscore’d compounds. With the filter applied this generated 12 candidates. This list contained 6 candidates with a perturbation score of 1.**

| **REFSETNAME** | **Tables count** | **Sum-Sigs** | **perturb stabil** | **SetScore** | **SetSize** |
| --- | --- | --- | --- | --- | --- |
| Wortmannin | 11 | 11 | 1 | -0.315291774 | 18 |
| Genistein | 11 | 11 | 1 | -0.386470205 | 17 |
| PHA-00745360 | 11 | 11 | 1 | -0.507332181 | 8 |
| Fludrocortisone | 11 | 11 | 1 | -0.562237696 | 8 |
| Alprostadil | 11 | 11 | 1 | -0.404307805 | 7 |
| Diethylstilbestrol | 11 | 11 | 1 | -0.519842748 | 6 |
| Estradiol | 11 | 10 | 0.909090909 | -0.192112987 | 37 |
| Etiocholanolone | 11 | 9 | 0.818181818 | -0.507999491 | 6 |
| Monorden | 11 | 8 | 0.727272727 | -0.215758977 | 22 |
| Paclitaxel | 11 | 7 | 0.636363636 | -0.298387833 | 6 |
| CP-320650-01 | 11 | 5 | 0.454545455 | -0.397394349 | 8 |
| Finasteride | 11 | 5 | 0.454545455 | -0.459616521 | 6 |
| Other data omitted.... |  |  |  |  |  |
